# Cytomembrane nanovaccines show therapeutic effects by mimicking tumor cells and antigen presenting cells

**DOI:** 10.1038/s41467-019-11157-1

**Published:** 2019-07-19

**Authors:** Wen-Long Liu, Mei-Zhen Zou, Tao Liu, Jin-Yue Zeng, Xue Li, Wu-Yang Yu, Chu-Xin Li, Jing-Jie Ye, Wen Song, Jun Feng, Xian-Zheng Zhang

**Affiliations:** 1 Key Laboratory of Biomedical Polymers of Ministry of Education, Department of Chemistry, Wuhan University, Wuhan, 430072 P.R. China; 2 The Institute for Advanced Studies, Wuhan University, Wuhan, 430072 P.R. China

**Keywords:** Cancer, Nanoscience and technology

## Abstract

Most cancer vaccines are unsuccessful in eliciting clinically relevant effects. Without using exogenous antigens and adoptive cells, we show a concept of utilizing biologically reprogrammed cytomembranes of the fused cells (FCs) derived from dendritic cells (DCs) and cancer cells as tumor vaccines. The fusion of immunologically interrelated two types of cells results in strong expression of the whole tumor antigen complexes and the immunological co-stimulatory molecules on cytomembranes (FMs), allowing the nanoparticle-supported FM (NP@FM) to function like antigen presenting cells (APCs) for T cell immunoactivation. Moreover, tumor-antigen bearing NP@FM can be bio-recognized by DCs to induce DC-mediated T cell immunoactivation. The combination of these two immunoactivation pathways offers powerful antitumor immunoresponse. Through mimicking both APCs and cancer cells, this cytomembrane vaccine strategy can develop various vaccines toward multiple tumor types and provide chances for accommodating diverse functions originating from the supporters.

## Abstract (web summary)

Cancer vaccines often fail to generate clinically relevant effects. Here, the authors generate a nanosized cytomembrane vaccine based on fusion between dendritic cells and cancer cells, and show them to activate anti-tumor immune responses via their antigen presenting and T-cell activating functions.

## Introduction

Training the autologous immune system to detect and eliminate systemic tumors, is emerging as a promising modality for cancer prevention and therapy^1,2^. Immune systems can detect the aberrant mutation via recognizing the specific antigens expressed by abnormal or transformed cells. However, cancer can develop various immune evasion mechanisms to delay, reduce, or even stop specific and non-specific immune attacks. For instance, cancer cells actively downregulate tumor antigens on cytomembranes to evade immune detection. To address this issue, the immunosurveillance toward oncogenesis and the immunoresponse of tumor-specific cytotoxic T-cells have to be reinforced by external stimuli, such as cancer vaccines^3,4^. Cancer vaccines are also an effective combinational partner with traditional oncotherapy approaches for better anticancer performances^5–7^.

Dendritic cells (DCs) play critical roles in inducing immune responses of T cells against pathogens and malignant cells^8,9^. DCs are specialized in taking up tumor antigens, processing and presenting tumor antigens in the form of antigen peptides-major histocompatibility complex (pMHC) on cytomembranes^10^. Thereafter, as the professional antigen-presenting cells (APCs), matured DCs prime different subsets of naive antigen-specific T cells to accomplish attacks to tumor cells. Based on the understanding about the cellular mechanism of this general immunoactivation process, the most popular cancer immunotherapy practice has been to immunize patients against tumor antigens through elevating the T cell-mediated immunity in quantity and/or quality by means of antigen/adjuvant vaccines and DC vaccines^11–14^. Overall, the majority of tumor vaccines are directed against a single antigen target. Despite a large number of vaccines have been tested so far, most of them were unsuccessful in eliciting clinically relevant effects due to the immune resistance along with tumor development and the tumor heterogeneity. Generally, high levels of tumor antigens within cancer vaccines are required to reach the threshold for T cell recognition, thereby breaking immunological tolerance but also amplifying the risk of immune-related adverse events due to the lack of complete tumor-specificity. Cancer cells bear a broad category of antigens including the tumor-associated and the tumor-specific. An important challenge is how to identify among the tumor antigens one or few that are the best with high tumor specificity^15,16^. Unfortunately, the known types of tumor antigens are very few so far. In addition, the cooperation among different antigens on cancer cell membranes may contribute to the activation of APCs^17^. Therefore, a rational choice to maximize tumor-specific immunoresponse may be to simulate the innate immunoactivation mechanism by introducing more types of tumor antigens into vaccines^18^. Nevertheless, this concept is significantly challenged by the limited availability of tumor antigen types. Moreover, the integration of multiple tumor antigens into one system is hardly accessible since the fabrication process is too expensive and effort-costing.

Cancer cell membranes have been recently proposed as cytomembrane vaccines^19^. However, a large proportion of tumor antigens may not be expressed on cytomembranes but included inside cancer cells. Furthermore, the downregulated expression of tumor antigens on cancer cell membranes for blunting immune systems hampered the success of this biological approach^20,21^. Researchers ex vivo fed DCs with the antigen-encoding DNA or mRNA to compel DC to express certain tumor antigen on cell surface^22,23^, providing artificially programmed APCs as cell-typed vaccines. This method was limited by the low expression of pMHC and difficult storage of APCs prior to usages. A cellular vaccine was generated based on the fusion between DCs and cancer cells, which offered hybrid cells that shared a unified cytoplasm but preserved the identity of dual nuclei^24,25^. It is noted that such a fused configuration induced the processing of the whole tumor antigens, including the known and the unidentified, and the immunological co-stimulatory molecules (e.g., B7 family members) on cytomembranes^26,27^. In addition, the fused cells (FCs) acquire the enhanced lymph node homing capability^28^, favoring antigen presentation to T cells in lymph nodes. Nevertheless, the application of this means suffers from carcinogenic risks and low survival rate of FCs.

**Fig. 1:**
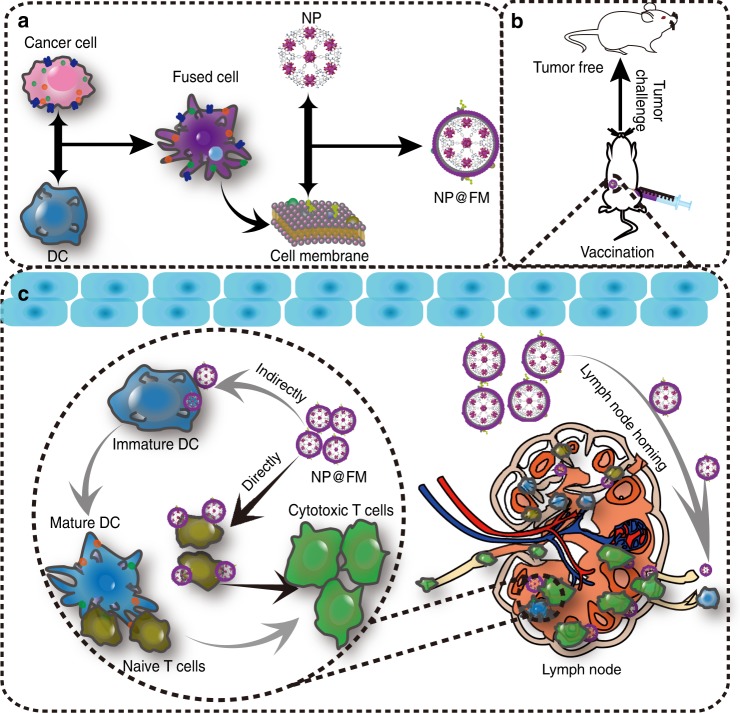
Schematic illustration of MOF@FM for tumor prevention. **a** Preparation of MOF@FM. **b** Vaccination of MOF@FM for tumor prevention. **c** Mechanisms of MOF@FM inducing immune responses

This study intends to engineer biologically derived tumor-specific vaccines, merely relying on the cytomembranes (FMs) of the FCs from DCs and tumor cells (Fig. 1). Nanoparticles (NPs) are incorporated as the supporter of FMs to provide nanosized vaccines (NP@FM) in view of the well-known merits associated with NPs, such as the long-circulating duration and the passive targeting at tumors^29,30^. NP@FM possesses better biosafety, easier large-scale fabrication, and longer storage compared with cellular vaccines because of the exclusion of genetic materials. Due to the similarity in outer membranes, NP@FM inherits and even amplify the interfacial biofunctions of the parent two cell lines, such as the lymph node-homing capacity of DCs. NP@FM is expected to confer not only the antigen-presenting functionality of APCs owing to the high expression of the whole tumor antigens presented as pMHC, but also a continuous source of endogenous tumor antigens derived from cancer cell membrane fragments, which can be recognized by DCs for maturation stimulation followed by the induction of T cell activation^31^. The combination of these two pathways (direct T cell activation and indirect DC-to-T activation) can offer powerful immune responses against cancer cells. The inclusion of whole tumor antigens within NP@FM may enhance the immune specificity to cancers. We hope this cytomembrane vaccine strategy can potentially develop the next generation of cancer vaccines toward multiple tumor types. To prove this concept, a fluorescent metal-organic framework (MOF) is used as the NP model for imaging purpose. Apparently, other supporter excipients with different characters (e.g., size and morphology) and diverse functions (e.g., therapeutics and vaccine adjuvants) can be freely selected for the complex requirements.

## Results

### Characterization of NP@FM NPs

Murine mammary carcinoma (4T1) cells were used here as the typical cancer cells. According to the reported method, the FCs were obtained on the basis of the polyethylene glycol (PEG) stimulated fusion of DCs and 4T1 cells^32^. Before fusion, 4T1 cells were treated with ethanol to make them express eat-me signals (e.g., calreticulin and immunostimulatory factors) on the surface^33^. The ethanol-treated 4T1 cells and DCs were then mixed at a ratio of 1:2 for cell fusion in the phosphate buffer solution (PBS) containing 50 wt% PEG (MW = 4000) and 10 wt% dimethyl sulfoxide (DMSO). The fusion process lasted for 2 min at 38 °C, then the cells were harvested and washed with the RPMI 1640 medium, and continuously cultured under normal condition for 6 days to enable the sufficient production of pMHC, co-stimulatory molecules, lymph node homing receptors (C–C chemokine receptor type 7, CCR7) on the FM^12^.

**Fig. 2:**
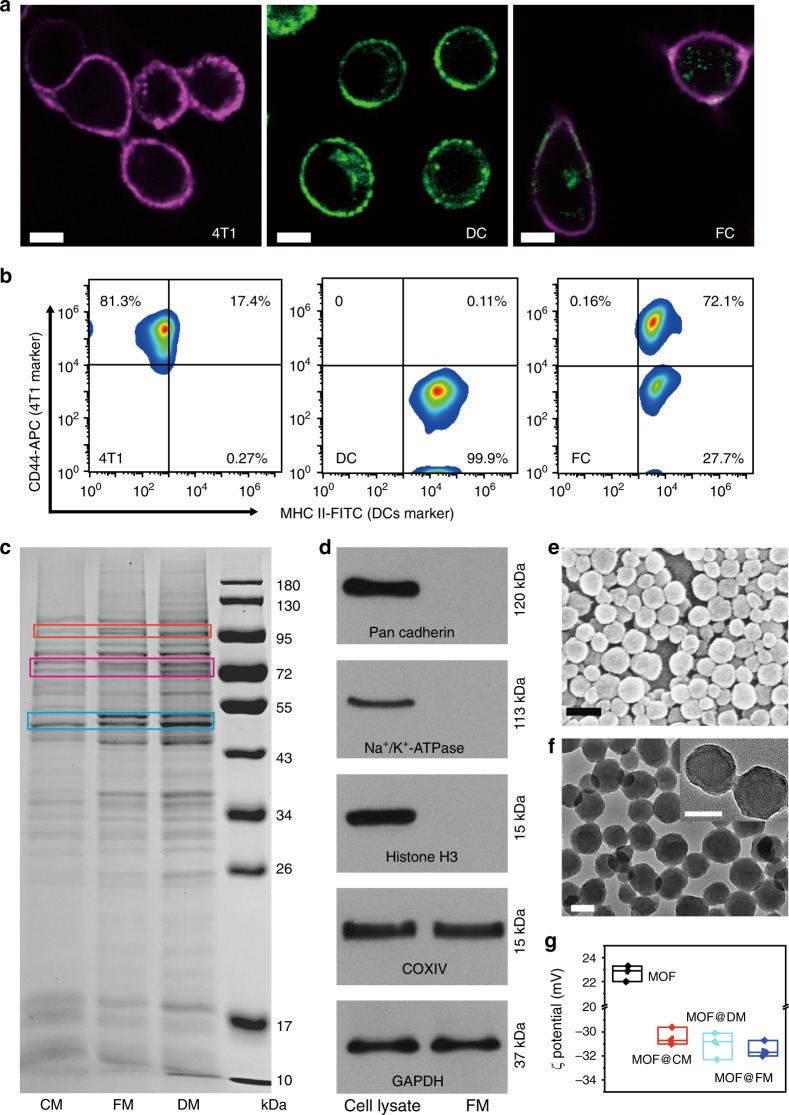
Characterization of FM and MOF@FM. **a** Fusion of DCs and 4T1 cells by CLSM observation over red fluorescence of anti-CD44-APC antibody marked 4T1 and green fluorescence of anti-MHC II-FITC antibody labeled DCs, and the double-labeled FCs. Scale bar = 10 μm. **b** Flow cytometric analyses of anti-CD44-APC antibody marked 4T1 cells, anti-MHC II-FITC antibody labeled DCs and the double-labeled FCs. **c** SDS-PAGE protein analyses of CM, DM, and FM. **d** Western blotting analysis of membrane-specific and intracellular protein markers in the FCs lysate and FM. **e** SEM image of MOF. Scale bar = 200 nm. **f** TEM images of MOF@FM. Scale bar = 100 nm. **g** Zeta potentials of MOF, MOF@CM, MOF@DM and MOF@FM. Measurements were taken from distinct samples (*n* = 3). Source data are provided as a Source Data file

Because the successful fusion of DCs and 4T1 cells was crucial to the pMHC expression on the cytomembranes of FCs, the cell fusion was first investigated. 4T1 cells were stained with Hoechst 33342 (blue fluorescence, nuclear dye) and DCs were marked with a cell membrane dye of 3,3′-dioctadecyloxacarbocyanine perchlorate (DiO, green fluorescence). The harvested FCs were clearly observed with the blue nuclear and green membrane by confocal laser scanning microscopy (CLSM) (Supplementary Fig. 1). Considering the possible interference originating from the diffusion of the nonspecific fluorescence dyes, we further used specific antibodies of cell markers to validate the successful fusion. When 4T1 cells were marked with magenta fluorescent anti-CD44-APC and DCs were labeled with green anti-MHC II-FITC^34–37^, there apparently appeared white fluorescence (representing the overlap of magenta and green fluorescence) in the FC cytomembranes (Fig. 2a). Based on the individual antibody labeling toward DC and 4T1 cells, the data of flow cytometry were obtained, which agreed well with the CLSM observation (Fig. 2b). These results demonstrate the successful fusion of DCs and 4T1 cells. The protein ingredients of the 4T1 membrane (CM), DC membrane (DM), and FM were analyzed through sodium dodecyl sulfate-polyacrylamide gel electrophoresis (SDS-PAGE). It is found that almost all the membrane proteins of CM and DM can be detected in FMs. Meanwhile, FMs displayed some new bands (marked with box) that seemed to not belong to either CM or DM (Fig. 2c), suggesting the expression of new proteins on FMs during cellular fusion. The clinical translation of cancer cells as therapeutics/delivers faces an important barrier because of the cancerogenic risk arising from the genetic materials of cancer cells. The obtained FMs were therefore analyzed with western blotting toward a series of protein markers in FC cells^38,39^. The result shows the good preservation of Pan Cadherins and Na^+^/K^+^-ATPase, both as plasma membrane-specific markers. Conversely, the typical intracellular protein markers, Histone H3, Cytochrome C oxidase subunit 4 isoform 1 (COX IV), and glyceraldehyde-3-phosphate dehydrogenase (GAPDH), respectively in the nucleus, mitochondria, and cytosol, didn’t appear on FMs, manifesting the minimal cancerogenic risk (Fig. 2d). Meanwhile, Hoechst 33342 was used to stain nucleic acid as shown in Supplementary Fig. 2. Negligible blue fluorescence appeared in FMs, which indicated that FMs hardly contained nucleic acid.

PCN-224 MOF, a fluorescent NP, was used here for imaging purpose^40^. MOF@FM was prepared by cloaking MOF with FMs under ultrasound in ice bath^41^. The nanoscale morphology of MOFs and MOF@FMs was clearly observable by scanning electron microscopy (SEM) (Fig. 2e). Transmission electron microscopy (TEM) reflects the core-shell structure of MOF@FM with a uniform cell membrane shell at about 10 nm in thickness (Fig. 2f). The uncloaked MOFs had a mean hydrodynamic diameter (*D*_h_) at about 145.6 nm with a narrow size distribution (Supplementary Fig. 3) and a positive charge potential (ζ) at 24.5 mV (Fig. 2g). Coating FM to MOF surface led to a subtle increase of *D*_h_ and a charge reversal of ζ potential. These findings manifest the successful coating of FM on MOF supporter. As the controls, the 4T1 cell membrane coated MOF@CM and the DC membrane coated MOF@DM were prepared in the same manner. Both of them showed similar *D*_h_ and ζ with MOF@FM. Of note, *D*_h_ of MOF@FM in the medium containing 10% serum remained steady over 7 days, as contrary to the marked increase of D_*h*_ observed for uncoated MOFs (Supplementary Fig. 4)^42,43^. This result suggests that the cytomembrane coating could largely enhance the serum-conditioned stability of nano-supporters, which certainly favors the in vivo application^44,45^. The similarity in the UV–Vis absorbance between MOF and MOF@FM indicates that the membrane coating insignificantly affects the optical property of MOFs (Supplementary Fig. 5). The biocompatibility of MOF@FM in vitro was examined in cancerous 4T1 cells (Supplementary Fig. 6a) and normal murine fibroblast (3T3) cells (Supplementary Fig. 6b) by 3-(4,5-dimethyl-2-thiazolyl)- 2,5-diphenyl-2-H-tetrazolium bromide (MTT) assay. The three kinds of membrane-cloaked MOFs and the uncloaked MOF exhibited minimal cytotoxicity in the tested cell lines at a high MOF concentration of 100 μg mL^−1^, indicating the good biocompatibility in cellular levels.

### In vitro immunoresponse of NP@FM NPs

**Fig. 3:**
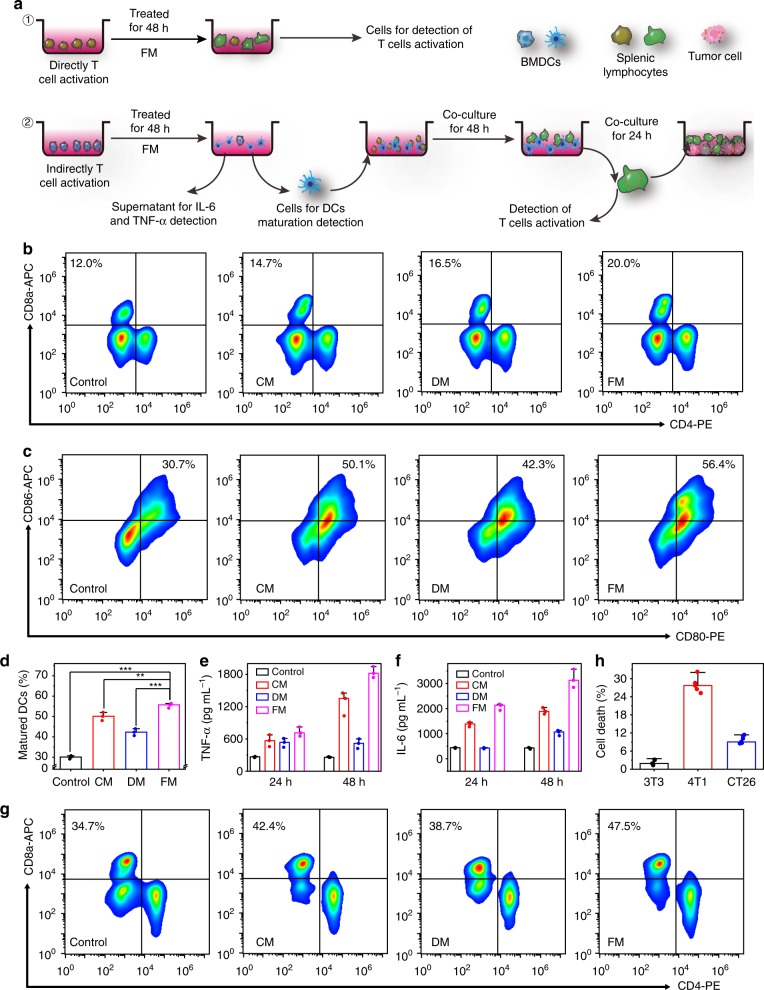
In vitro immune cells activation by cytomembrane nanovaccines. **a** Illustration of the in vitro immune experiments. **b** Flow cytometric analyses of the expression of CD8 and CD4, the markers for T cells activation, after in vitro incubation of T cells with CM, DM, and FM for 48 h. **c** Flow cytometric quantification of the expression of CD80 and CD86 (the markers for DC maturation) after in vitro incubation of DCs with CM, DM, and FM for 48 h. **d** The percentage of DC maturation. The mean values and s.d. were presented and measurements were taken from distinct samples (one-way ANOVA; **p < 0.01, ***p < 0.001, *n* = 3). **e** Secretion of TNF-α in DC suspensions measured by ELISA kit. The mean values and s.d. were presented and measurements were taken from distinct samples (*n* = 3). **f** Secretion of IL-6 in DC suspensions measured by ELISA kit. The mean values and s.d. were presented and measurements were taken from distinct samples (*n* = 3). **g** Quantification of the expression of CD8 and CD4, the makers for T cell activation, after in vitro incubation of the above-pretreated DCs with splenic lymphocytes for 48 h. **h** In vitro cytotoxicity of the above-activated T lymphocytes to 3T3, 4T1, and CT26 cells. The mean values and s.d. were presented and measurements were taken from distinct samples (*n* = 5). Source data are provided as a Source Data file

Provided that tumor antigens could be processed and expressed on FMs during cellular fusion, FMs could present tumor antigens to T cells and directly active T cells owing to the partial inclusion of DC’s cytomembrane fragments in FMs. Like tumor cells, FMs can be recognized and taken up by DCs and consequently, the matured DCs can serve as APCs to present antigens to T cells. The demonstration of these two of direct and indirect pathways are illustrated in Fig. 3a. To avoid the interference of MOF’s fluorescence on the immune fluorescence staining, the following in vitro experiments were conducted by using the cytomembranes (CM, DM, and FM) alone to investigate immune responses. Because CD8+ cytotoxic T lymphocytes (CTLs) are the main force to kill cancer cells in our immune design^46,47^, we measured the expression of CD8 on the cytomembrane of CD3+ T cells (from mouse splenocytes) via flow cytometry to investigate the direct pathway (Fig. 3b and Supplementary Fig. 7a). After 48 h coincubation, the percentage of CD8+ CTLs was dramatically increased. In comparison, much less increment was observed in the DM and CM treated groups. The result indicates that FMs were more powerful to activate T cells into CTLs than CMs and DMs. In the fusion process, DCs can capture and process the tumor antigens of tumor cells, and then present a whole array of tumor antigens in the form of pMHC to T cells with the help of upregulated co-stimulatory molecules. Compared with the other two cytomembranes, therefore, FMs induced the activation of T cells at a higher level. Although CM contained innate tumor antigens, its efficacy of T cell activation seemed to be similar or even lower than that of DMs. This finding is possibly related to the specific recognition of DCs by T cells.

As to the indirect DC-to-T immunoactivation pathway, we first investigated the cell uptakes of the different cytomembrane cloaked MOFs by bone marrow-derived DCs (BMDCs). In terms of the internalization efficiency in BMDCs, it is evident that BMDCs preferred MOF@CM and MOF@FM to MOF@DM (Supplementary Fig. 8), owing to the specific recognition of DCs to the tumor antigens in both CMs and FMs. We next assessed the in vitro immunostimulatory activity of DCs after the treatment with three kinds of cytomembranes. In principle, the expression of special co-stimulatory molecules (CD80 and CD86) would be enhanced as the result of the induced DC maturation^48,49^. The maturity can be hence evaluated based on the measurement of these co-stimulatory molecules on DCs upon the exposure to the cytomembranes. Along this line, CM, DM, and FM were co-cultured with BMDCs for 48 h before the measurement. The percentage of CD80+ and CD86+ DCs after the treatment with FMs was obviously higher than that of other controls (Fig. 3c, d and Supplementary Figs. 7b, 9). It is interesting to point out that FM induced DC maturation more effectively than CM although they both contain tumor antigens. We assume that this discrepancy may have an association with the expression of whole tumor antigens in the pMHC form within FMs, which may contribute to the enhanced DC maturation after the internalization by DCs. Meanwhile, the immune-related cytokines secreted by DCs, including tumor necrosis factor α (TNF-α) and interleukin 6 (IL-6), which are also important indicators of DC maturation^50,51^, were measured by enzyme-linked immunosorbent assay (ELISA). It was found that the level of TNF-α and IL-6 in the FM-treated DCs was increased with incubation time and always remained higher than those in the DM and CM treated groups (Fig. 3e, f). Relatively, CM treatment led to higher expression of the immune-related cytokines in the DCs than DM treatment. All the above results demonstrate that compared with DMs and CMs, FMs were more subject to the specific recognition by BMDCs, resulting in more effective induction of BMDC maturity. DCs can discern antigens and then process them into antigen peptides in the form of pMHC during migration from peripheral tissues to nearby draining lymph nodes, where they present the pMHC to T cell receptors (TCR) for T cell activation to kill tumor cells^52^. The activation of T cells by the above-activated DCs was thus evaluated via flow cytometry. When T cells were coincubated with the FM-treated DCs for 48 h, the percentage of CD3 + CD8+ CTLs was higher than the other control groups (Fig. 3g). The activated T cells by the different cytomembranes treated DCs were cultured with 4T1 cells to measure cell cytotoxicity, respectively (Supplementary Fig. 10). The cell-toxic effect in group of FM was much higher than other groups, indicating the strong immune activation of FM. To study the immunologic specificity of FM to 4T1, the FM activated T cells were then cultured with 3T3, 4T1, and CT26 (mouse colon cancer cells) at a number ratio of 10:1 (T cells verse target cells) for 24 h for lactate dehydrogenase assay, respectively (Fig. 3h). The cell-toxic effect in 4T1 cells was much stronger than those in the other cells, and minimal cell-toxic was observed in 3T3 group, indicating the immunologic specificity of the FM to 4T1. All the results agree well with each other in terms of the indirect activation pathway of T cells.

**Fig. 4:**
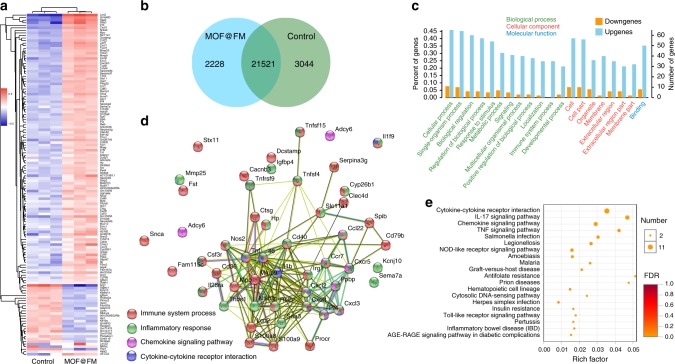
Transcriptome analyses of MOF@FM induced DCs. **a** Heat map showing significantly upregulated and downregulated genes of DCs after MOF@FM treatment (fold change ≥ 2 and *p* < 0.05). **b** Venn diagram of a primary transcript between MOF@FM and PBS. **c** The changes of genes associated with biological process, cellular component, and molecular functions based on GO annotation. **d** The analyses of functional interaction network of MOF@FM regulated genes by using the Search Tool for the Retrieval of Interacting Genes/Proteins (STRING) algorithm. **e** KEGG pathway analysis for immune response associated different genes. Measurements were taken from distinct samples (*n* = 3). Source data are provided as a Source Data file

To deeply investigate the immunoresponse mechanism of DCs to MOF@FM, the transcriptome of DCs was conducted after MOF@FM treatment for 48 h (the transcriptome analysis was assisted by Majorbio). As shown in Fig. 4a, 110 upregulated genes and 24 downregulated genes were identified (fold change ≥ 2 and *P* < 0.05) in MOF@FM treated group relative to PBS control. Results of both cluster analysis and principal component analysis revealed the significant differences between PBS and MOF@FM treated groups. The Venn diagram shown in Fig. 4b indicated that there occurred significant discrepancy of several primary transcripts between PBS and MOF@FM groups. On the basis of the Gene Ontology (GO) analysis, it was found that the genes related to biological process, cellular component and molecular function, were mostly upregulated profoundly in the MOF@FM group (Fig. 4c). The upregulation of the genes related to immune system process evidenced that MOF@FM could induce immune responses. The upregulation of the genes associated with binding ought to be responsible for the migration and location of DCs in the lymph node. From the protein-protein interactions network analysis deduced from differential gene expression in MOF@FM group, we identified four kinds of immune-associated functional protein networks, which were involved in the immune system process, inflammatory response, chemokine signaling pathway and cytokine-cytokine receptor interaction, respectively (Fig. 4d). This result is reasonably understandable. For instance, in immune system process, matrix metalloproteinase-9 (Mmp9) mediates transmigration of inflammatory leukocytes across the basement membrane, which is important for DC recruitment to inflammatory tissue, e.g., tumor^53^. TNF-α and IL-6 play an essential role in the immune system process. Accordingly, the inflammatory response and cytokine-cytokine receptor interaction are clearly shown in Fig. 4d and DCs secreted more TNF-α and IL-6 after MOF@FM treatment (Fig. 3e, f))^54,55^. Kyoto Encyclopedia of Genes and Genomes (KEGG) pathway analysis (Fig. 4e) reflects the comprehensive immune response of BMDCs, indicating the major activation pathways including cytokine-cytokine receptor interaction, chemokine signaling pathway, and TNF signaling pathway. The activation pathways may have an association with not only the MOF@FM activated DC maturation but also the infection between mature and immature DCs^56,57^.

### In vivo NP@FM as a vaccine for tumor prevention

**Fig. 5:**
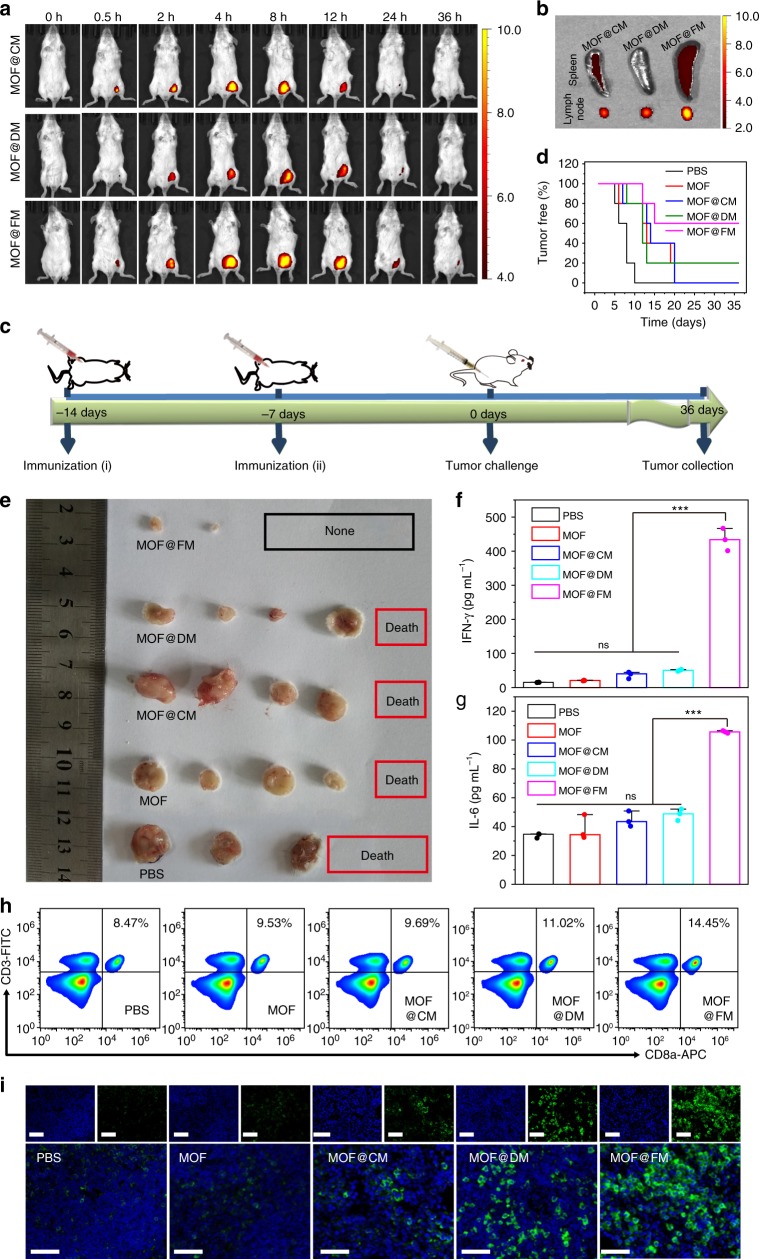
MOF@FM as a vaccine for tumor prevention. **a** In vivo fluorescence imaging at the indicated time points after the subcutaneous injection of samples. **b** Ex vivo fluorescent images of lymph node and spleen at 36 h after subcutaneous injection. **c** Illustration of the experiment design. Healthy mice were immunized twice in every week by subcutaneous injection and tumor challenge at 7 d after the last immunization. **d** Percentage of tumor-free mice after tumor challenge. **e** Photos of harvested tumors at 36 d after tumor challenge. **f** Levels of secreted IFN-γ in mice serum measured by ELISA kit. The mean values and s.d. were presented and measurements were taken from distinct samples (one-way ANOVA; ns not significant, ***p < 0.001, *n* = 3). **g** Levels of secreted IL-6 in mice serum measured by ELISA kit. The mean values and s.d. were presented and measurements were taken from distinct samples (one-way ANOVA; ns: not significant, ***p < 0.001, *n* = 3). **h** Flow cytometric quantification of CD3 and CD8 expressed by splenic lymphocytes at 7th day after twice immunizations. **i** Immunofluorescence observation over CD8 in the draining lymph node; Scale bar = 50 μm. Source data are provided as a Source Data file

Encouraged by the in vitro results, the potential of MOF@FM as the vaccine in tumor prevention was evaluated in vivo. It is universally accepted that many vaccine systems have the ability to form an antigen depot at the administration site^58–60^. This effect can extend the exposure time of antigens to the immune system, thereby facilitating antigen capture by the immunity system for powerful immune responses. To explore the antigen depot effect, mice were injected subcutaneously with MOF@CM, MOF@DM and MOF@FM at the left groin near the draining lymph node, respectively. Vaccine persistence at injection sites was inspected by an in vivo imaging system at the predetermined time points. Lymph node homing refers to the migration of matured DCs from peripheral non-lymphoid tissues into secondary lymphoid tissues after the uptake of antigens. The progressively mature DCs are out of contact with epithelial cells or other cells and upregulate the expression of MHC II molecules, costimulatory molecules, adhesion molecules, and chemokine receptors (i.e. CCR7), which enhance the ability of DCs for the migration and location in the lymph node. Considering that the fusion of DCs and cancer cells is accompanied by DC maturation, FMs are bound to contain lymph node homing molecules and thus share the lymph node homing ability. As shown in Fig. 5a, the fluorescent signal of MOF@FM was always higher than that of MOF@DM and MOF@CM. The better retention effect corresponded well to the effect of lymph node-tropic migration and location after DC maturation (Fig. 5b). The ex vivo fluorescence observation over spleen and draining lymph node, the major immune organs, reconfirmed this conclusion. MOF@FM afforded much stronger fluorescence signals in these two organs than MOF@CM and MOF@DM did.

To investigate the in vivo efficacy of MOF@FM vaccine against 4T1 tumors, MOF, MOF@CM, MOF@DM and MOF@FM were immunized at the left groin of healthy BALB/C mice through subcutaneous injection for twice at the one-week interval, respectively. Seven days later, the BALB/C mice were inoculated with 5 × 10^4^ 4T1 cells subcutaneously at right hind leg (Fig. 5c). Palpable tumors were found in all the mice in the PBS group at 10 day after tumor challenge. In comparison, the pretreatments with membrane-coated nanovaccines prolonged tumor-free time at different degrees. The percentage of tumor-free mice reached as high as 60% for MOF@FM at 36 day, while that dropped to zero in the control pretreated with MOF@CM and 20% with either MOF or MOF@DM (Fig. 5d). The tumors were collected at 36 day. The photos of tumors indicate that MOF@FM could prevent tumor occurrence very effectively (Fig. 5e). Interestingly, MOFs alone seemed to be able to induce a moderate immune response when compared with the PBS group, which has been not reported to our knowledge. The possible reason may be ascribed to the enhanced immunoresponse due to the foreign intruders. We are surprised that MOF@CM had minimal effect to prevent tumor proliferation while MOF@DM showed evident tumor-preventive effect. The former may relate to the immunoescape mechanisms of cancer cells, such as the expression of program death-ligand 1 (PD-L1) in CM and the downregulated level of tumor antigens^61,62^. However, the tumor-preventive effect of MOF@DM was hard to understand at present though this finding was interesting.

As aforementioned, the secretion of immunostimulatory cytokines is an important indicator of the immune responses. IL-6 and interferon-γ (IFN-γ) are important immunostimulatory cytokines, which help promote the maturation of DCs and the differentiation of CD8+ T cell precursors into fully cytotoxic CTLs^63,64^. At the seventh day after immunization, the mice were sacrificed and the serums, spleens and the draining lymph nodes were collected for analyses. The secretion level of IL-6 and IFN-γ in serum was determined by ELISA, respectively. As shown in Fig. 5f, g, MOF@FM evoked the highest secretion of IFN-γ and IL-6 among all the tested groups. The level of IFN-γ and of IL-6 in MOF@FM group was increased by about 27 and 3 times compared with the PBS group, respectively. Compared with PBS control, the corresponding secretion levels in MOF@DM and MOF@CM groups were improved moderately. After the mice were sacrificed, the splenocytes of the treated mice were also collected and stained with anti-CD3-FITC and anti-CD8-APC to differentiate CTLs (Fig. 5h and Supplementary 7c). The vaccination with MOF@FM led to a significant increment of CD3+ CD8+ CTLs compared with PBS control. The corresponding percentage of CTLs in MOF@FM group reached 14.45%, which was higher than that of PBS (8.47%), MOF (9.53%), MOF@CM (9.69%), and MOF@DM (11.02%) groups. Furthermore, the draining lymph nodes were processed by immunofluorescent staining with CD8 antibody, and the strongest fluorescence appeared in the group of MOF@FM (Fig. 5i). All these results suggest the promise of MOF@FM in tumor-specific immunity.

Acceptable bio-safety of biomaterials is an essential requirement before their translation into clinical trials. The major organs including heart, liver, spleen, lung and kidney were harvested at 36 day after tumor inoculation for H&E assay. There appeared no obvious physiological abnormalities in these organs after the subdermal administration of all the tested samples (Supplementary Fig. 11). Hemolysis test was carried out to evaluate the compatibility of MOF@FM with blood erythrocyte, indicating the minimal hemolytic reaction (Supplementary Fig. 12). These results confirm the minimal systematic toxicity of MOF@FM.

## Discussion

We have engineered a biologically derived nanovaccine (NP@FM) by wrapping the nano-supporter with the cytomembrane of FCs acquired from DCs and cancer cells. In addition to the lymph node homing ability, NP@FM displayed the antigen-presenting ability to activate T cells due to the presence of pMHC and co-stimulatory molecules, which were generated or upregulated during cellular fusion. Like tumor cells, NP@FM can be recognized by DCs for the induction of DC maturation followed by the T cell activation, owing to the inclusion of cancer cytomembrane fragments in FMs. The potential of NP@FM as a vaccine to resist tumor challenge was validated in vitro and in vivo. Based on the results, it is expected that this strategy of the cytomembrane vaccine can be applied for the preventive immunotherapy of multiple tumor types. Here, a fluorescent MOF was used as the NP model for imaging purpose. Apparently, other supporter excipients with different characters (e.g., size and morphology) and diverse functions (e.g., therapeutics, vaccine adjuvants) can be freely optioned for the complex requirements, such as the combinational therapy. This is assuredly an unparalleled advantage compared with adoptive cell vaccines. This study offers a general and versatile approach for the development of a class of cell-free vaccines.

## Methods

### Materials

Zirconyl chloride octahydrate (ZrOCl_2_·8H_2_O) and benzoic acid (BA) were purchased from Sinopharm Chemical Reagent CO., Ltd, (China). Tetrakis (4-carboxyphenyl) porphyrin (TCPP) was obtained from Key Laboratory of Biomedical Polymers of Ministry of Education & Department of Chemistry. Micro lactate dehydrogenase assay kit was purchased from and interleukin-4 (IL-4) were purchased from Beyotime Institute of Biotechnology (China). Dulbecco’s modified Eagle’s medium (DMEM), RPMI 1640 medium, fetal bovine serum (FBS), penicillin-streptomycin, and trypsin were obtained from BI Corp. Granulocyte-macrophage colony-stimulating factor (GM-CSF), Anti-CD44-APC (CAT # 103012, 0.25 μg per 10^6^ cells in 100 μL volume), Anti-MHC II-FITC (CAT # 109905, 0.25 μg per 10^6^ cells in 100 μL volume), anti-CD11c-FITC (CAT # 117306, 0.25 μg per 10^6^ cells in 100 μL volume), anti-CD80-PE (CAT # 104708, 0.5 μg per 10^6^ cells in 100 μL volume), anti-CD86-APC (CAT # 105012, 0.25 μg per 10^6^ cells in 100 μL volume), anti-CD3-FITC (CAT #, 100204, 0.5 μg per 10^6^ cells in 100 μL volume), anti-CD4-PE (CAT # 10408, 0.25 μg per 10^6^ cells in 100 μL volume), and anti-CD8a-APC (CAT # 100712, 0.25 μg per 10^6^ cells in 100 μL volume) antibodies were purchased from BioLegend, Inc (USA). ELISA kits of TNF-α, IL-6 and IFN-γ were purchased from 4A Biotech Co., Ltd. All reagents were directly used without purification unless specified mentioned.

### Characterization

TEM photos were gained from JEM-2100 (JEM Ltd., Japan). Hydrodynamic diameter and zeta potential were measured by dynamic light scattering (DLS) of Malvern Zetasizer ZEN3600. UV–vis absorbance was measured by UV–vis spectrophotometry Lambda 35 (Perkin-Elmer). The fusion of DC and 4T1 cells was performed by CLSM (PerkinElmer Ultra VIEW VoX). The flow cytometric analysis was performed by flow cytometer (BD Accuri C6). The DM, CM, and FM were dispersed in SDS buffer to examine their protein contents by SDS-PAGE gel electrophoresis.

### Cell line

4T1 cells, 3T3 cells, and CT26 cells were purchased from the China Center for Type Culture Collection.

### Generation of BMDCs

BMDCs were generated from the bone mesenchymal stem cells (BMSCs) by induced differentiation^65^. Briefly, the BMSCs were flushed from mouse marrow cavities of femurs and tibias and were cultured in the RPMI-1640 medium containing 20% FBS in the presence of recombinant GM-CSF (20 ng mL^−1^) and IL-4 (10 ng mL^−1^). After 6 days, BMDCs were harvested for further use.

### Methods for fusing of DC and 4T1 cells

4T1 cells were pretreated with 20% alcohol in an ice bath for 15 min to make 4T1 inactive. In a 50 mL centrifuging tube, the BMDCs and inactive 4T1 cells were mixed at a ratio of 2:1 and centrifuged at 500 g with brake and acceleration turn off for 10 min. In 60 s, a total of 1 mL pre-warmed 50% PEG (MW: 4000, w/w) and 10% DMSO was added dropwise to the centrifugal sedimentation with continuous and gentle stirring^66^. After 2 min of stewing in 38 °C, serum-free RPMI1640 was dripped slowly into the mixture until the overall volume reached 50 mL to end fusion. The solution was centrifuged and the FCs were re-suspended in RPMI 1640 containing 10% FBS, 1% antibiotics (penicillin-streptomycin, 10000 U mL^−1^) and IL-4 (10 ng mL^−1^). The FCs were cultured in a humidified atmosphere containing 5% CO_2_ at 37 °C for 6 days and the medium was changed every second day with RPMI 1640 containing 10% FBS, 1% antibiotics (penicillin-streptomycin, 10000 U mL^−1^) and IL-4 (10 ng mL^−1^).

### Preparation of FM

After FCs were cultured for 6 d, the cells were detached with a cell scraper to collected^67^. Then the collected cells were washed with cooled PBS (pH = 7.4) for twice. After that, the obtained cell pellets were further suspended in a hypotonic lysing buffer containing phenylmethanesulfonyl fluoride (PMSF) (Beyotime Institute of Biotechnology) and incubated in ice-bath for 15 min according to the manufacturer’s instructions. Afterward, the cells in the above solution were broken using a repeated freeze-thaw method for three times and further centrifuged at 700 × *g* for 10 min at 4 °C. The supernatant was further centrifuged at 14,000 × *g* for 30 min to collect the cracked cell membrane. The products of the cell membrane were lyophilized and stored at −80 °C. The lyophilized membrane materials are rehydrated in ultrapure water prior to use.

### Preparation of MOF

MOF was synthesized according to the method in reported literatures^68^. Briefly, TCPP (60 mg), ZrOCl_2_ (180 mg), and benzoic (1.68 g) were dissolved in 60 ml of DMF. After stirring for 5 h at 90 °C, the collected mixture was centrifuged at 10,000 × *g* for 30 min and thoroughly washed three times with DMF. The obtained MOF nanoparticles were preserved in DMF solution for storage. Before using MOF for experiments, the DMF solution was exchanged with ultrapure water by centrifugation.

### Preparation of FM coated MOFs

The MOF solution was added into the ultrapure water dispersion of FM with an equal weight of MOF and FM. The mixed solution underwent ultrasonic treatment in a cold water bath until the solution was transparent. The obtained MOF@FM nanoparticles were further purified by centrifugation to remove the free FM.

### Immune responses in vitro

To assess the maturation levels of BMDCs after different treatments, CM, DM and FM (40 μg mL^−1^) were incubated with BMDCs for 48 h. The cells were washed three times with PBS and subsequently stained with anti-CD11c-FITC, anti-CD80-PE, and anti-CD86-APC antibodies (BioLegend) for 30 min at 4 °C. After being washed with cold PBS, the cellular fluorescence was detected by flow cytometry (BD Accuri C6). All groups were analyzed in triplicate. Furthermore, to assess the activation levels of T lymphocytes, BMDCs were pre-treated as above mentioned. After 48 h, T lymphocytes were added to BMDCs at a ratio of 10 (BMDCs to T cells). After co-cultured for 48 h, the T lymphocytes were washed three times with PBS and subsequently stained with anti-CD3-FITC, anti-CD4-PE, and anti-CD8-APC antibodies (BioLegend) for 30 min at 4 °C. After being washed with cold PBS for thrice, the cellular fluorescence was detected by flow cytometry. The killing ability of MOF@FM activated immune cells to 3T3, 4T1 and CT26 (the ratio of activated splenic lymphocytes to target cells was 10:1) was conducted according to the instruction of micro lactate dehydrogenase assay kit.

To deeply investigate the immunoresponse mechanism of DCs to MOF@FM, the transcriptome of DCs was conducted. Identified genes with significant upregulation and downregulation were mapped (fold change ≥ 2 and *P* < 0.05). Based on GO annotation, the changes of genes associated with biological process, cellular component, and molecular functions were analyzed. To analyses of the functional interaction network, the Search Tool for the Retrieval of Interacting Genes/Proteins (STRING) algorithm was employed. immune response associated different genes were analyzed by KEGG pathway. All the analysis was based on the integrated cloud platform of I-Sanger (https://www.i-sanger.com/).

### Cytokines measurement

To measure the cytokine secreted by immune cells after stimulation, the suspensions of BMDCs culture media after stimulating with CM, DM and FM (40 μg mL^-1^) were collected at different time points (24 and 48 h) post stimulation. These samples were diluted to appropriate concentrations for further analysis. TNF-α and IL-6 were determined with corresponding ELISA kits according to the manufacturer’s protocol. All groups were analyzed in triplicate.

### Antigen persistence at the injection site

All of the animal experiments were conducted under protocols approved by the Institutional Animal Care and Use Committee (IACUC) of the Animal Experiment Center of Wuhan University (Wuhan, China). All mouse experimental procedures were performed in accordance with the Regulations for the Administration of Affairs Concerning Experimental Animals approved by the State Council of the People’s Republic of China. To monitor antigen persistence at the injection site in vivo, MOF@CM, MOF@DM, and MOF@FM at an equal amount of MOF (50 μL, 3.2 mg mL^−1^ per mouse) were injected to the inguinal region of mice, respectively. The in vivo living imaging was carried out with the IVIS imaging system at the predetermined time intervals post injection. The treated mice were sacrificed at 36 h postinjection and the spleen and lymph nodes nearby the injection site were harvested and imaged.

### Immunization and tumor cell challenge assay

BALB/C mice were divided into four experimental groups and immunized two times at an interval of 1 week by intradermal injections of with PBS, MOF@CM, MOF@DM, and MOF@FM at an equal amount of MOF (50 μL, 3.2 mg mL^−1^ per mouse). Seven days later after the last vaccination, 5 × 10^4^ 4T1 cells were transplanted subcutaneously into the right flank of mice. Then the location of the injected skin was monitored every day to evaluate the process of the tumor and the first day that the tumor could be macroscopically recorded as the tumor-free day.

### Immune response in vivo

To further evaluate the immune response in vivo, BALB/C mice were divided into four experimental groups and immunized two times at an interval of 1 week by intradermal injections of with PBS, MOF, MOF@CM, MOF@DM, and MOF@FM at an equal amount of MOF (50 μL, 3.2 mg mL^−1^ per mouse). Seven days later after the last vaccination, the peripheral blood which was isolated from mice was centrifuged to obtain the serum. Then the different cytokines in serum were quantitatively analyzed. Briefly, the IL-6 (IL-6, 4 A Biotech Co., Ltd) and IFN-γ (4 A Biotech Co., Ltd) release were detected by ELISA according to the protocol. The spleens were harvested and triturated to obtain a single cell suspension. The cells were filtered through 75 µm filters after washed twice and then the red blood cells were removed by using red blood cell lysis buffer (ACK lysis buffer). Then the T lymphocytes were incubated with anti-CD3-FITC, anti-CD8a-APC, and anti-CD4-PE antibodies after the Fc block of the cells and analyzed by flow cytometry. The lymph nodes were harvested and fixed with 4% paraformaldehyde, embedded with paraffin, and sliced up. After that, the CD3 and CD8 were stained. The images were obtained by an inverted fluorescence microscope.

### Statistical analysis

One-way analysis of variance (ANOVA) was used for multiple-group analysis. *p < 0.05, ***p < 0.01, ***p < 0.001. *p*-value of <0.05 was considered as statistically significant.

### Reporting summary

Further information on research design is available in the Nature Research Reporting Summary linked to this article.

## Supplementary information

Supplementary Information

Reporting Summary

Source Data

## Data Availability

The source data underlying Figs. 2c-d, g, 3d-f, h, 4a, 5f-g and Supplementary Figs 3, 4, 5, 6, 8, 10 and 12 are provided as a Source Data file. All the relevant data are available from the authors upon reasonable request. A reporting summary for this article is available as a Supplementary Information file.
