# Supplementary material for: Cytomembrane nanovaccines show therapeutic effects by mimicking tumor cells and antigen presenting cells
Source: Nat Commun. 2019 Jul 19;10:3199. doi: 10.1038/s41467-019-11157-1 (PMC6642123; doi:10.1038/s41467-019-11157-1)
Supplement: Supplementary file 1 — Supplementary Information [file 41467_2019_11157_MOESM1_ESM.pdf]

Cytomembrane nanovaccines show therapeutic effects by mimicking tumor cells and antigen presenting cells

Liu *et al.*

Table of contents

Supplementary Figure 1│CLSM images of 4T1, DC and FC....................S3

Supplementary Figure 2│Fluorescence images of cells, cell lysate and FMs with or without nuclease treatment after stained with Hoechst 33342...........S4

Supplementary Figure 3│Hydrodynamic sizes of MOF, MOF@CM, MOF@DM and MOF@FM..S5

Supplementary Figure 4│Stability of MOF and MOF@FM in water and serum..S6

Supplementary Figure 5│UV-vis spectra of FM, MOF and MOF@FM........S7

Supplementary Figure 6│In vitro cytotoxicity of MOF, MOF@CM, MOF@DM and MOF@FM.. ....S8

Supplementary Figure 7│Flow cytometry sequential gating strategies........S9

Supplementary Figure 8│Flow cytometry analysis of the uptake of BMDCs to MOF@CM, MOF@DM and MOF@FM and the corresponding mean fluorescence intensity (MFI) analysis. S10

Supplementary Figure 9│DCs maturation after treatment with CM, DM and FM, respectively..S11

Supplementary Figure 10│In vitro cytotoxicity of the CM, DM or FM-pretreated DCs activating T lymphocytes against 4T1 cells.......................................S12

Supplementary Figure 11│H&E staining of major organs at 36 d after tumor inoculation. Scale bar = 20 μm...S13

Supplementary Figure 12│Hemolytic assay of MOF, MOF@CM, MOF@DM and MOF@FM..S14

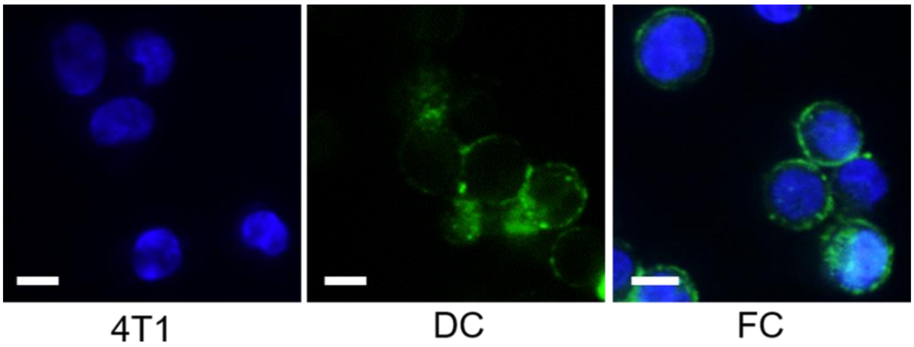

Supplementary Figure 1│CLSM images of 4T1, DC and FC. CLSM images of 4T1 marked with Hoechst 33342, DCs labeled with DiO, and FCs obtained from the fusion of dyes marked 4T1 and DCs. Scale bar = 16 μm.

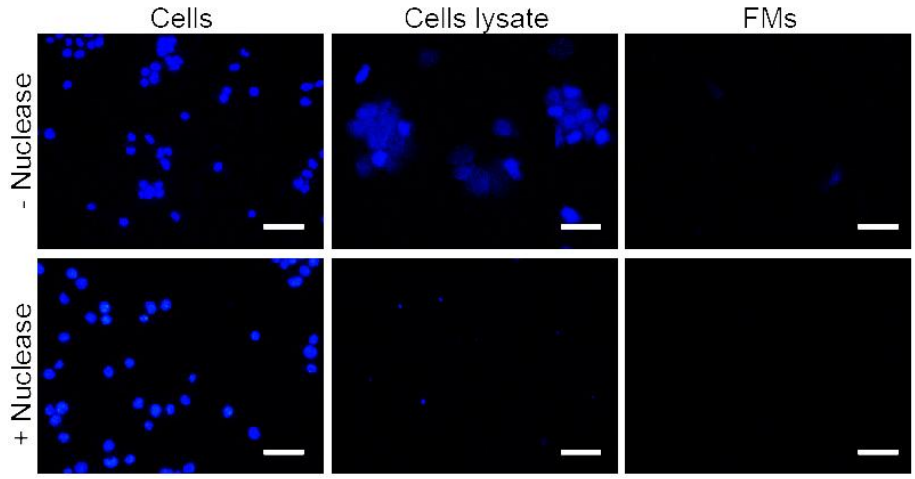

Supplementary Figure 2│Fluorescence images of cells, cell lysate and FMs with or without nuclease treatment after stained with Hoechst 33342. Scale bar = 50 μm.

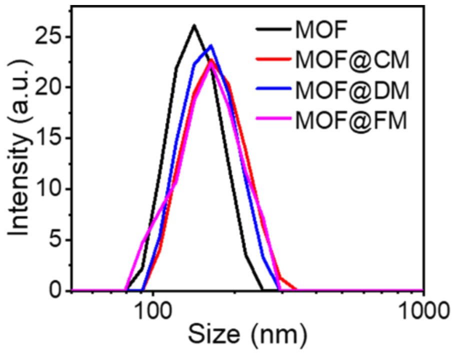

Supplementary Figure 3│Hydrodynamic sizes of MOF, MOF@CM, MOF@DM and MOF@FM. Dynamic light scattering (DLS) measurements of MOF, MOF@CM, MOF@DM and MOF@FM in water (pH = 7.4). Source data are provided as a Source Data file.

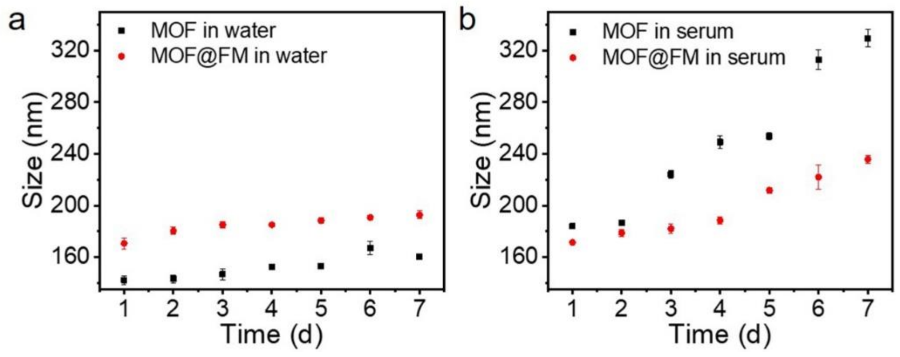

Supplementary Figure 4│Stability of MOF and MOF@FM in water and serum. Hydrodynamic size changes with time of MOF and MOF@FM in water (**a**) and serum (**b**), respectively. The mean values and s.d. were presented and the same sample was measured repeatedly (n = 3). Source data are provided as a Source Data file.

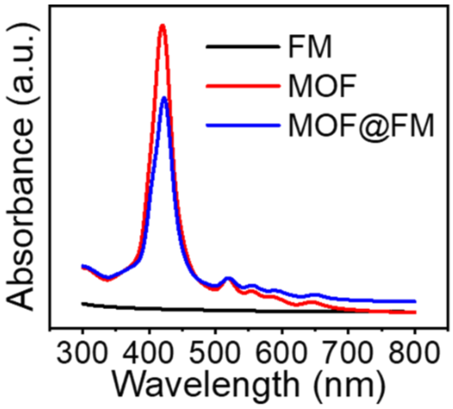

Supplementary Figure 5│UV-vis spectra of FM, MOF and MOF@FM. Source data are provided as a Source Data file.

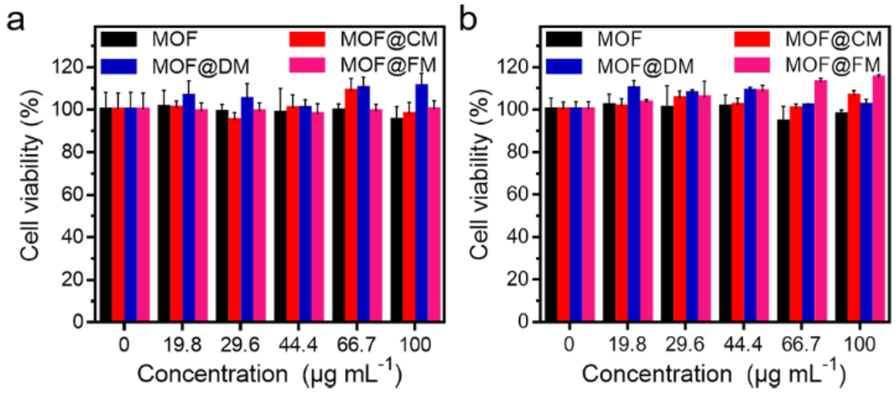

Supplementary Figure 6 | In vitro cytotoxicity of MOF, MOF@CM, MOF@DM and MOF@FM. In vitro cytotoxicity to 4T1 (**a**) and 3T3 (**b**) after treatment with MOF, MOF@CM, MOF@DM and MOF@FM. The mean values and s.d. were presented and measurements were taken from distinct samples (n = 5). Source data are provided as a Source Data file.

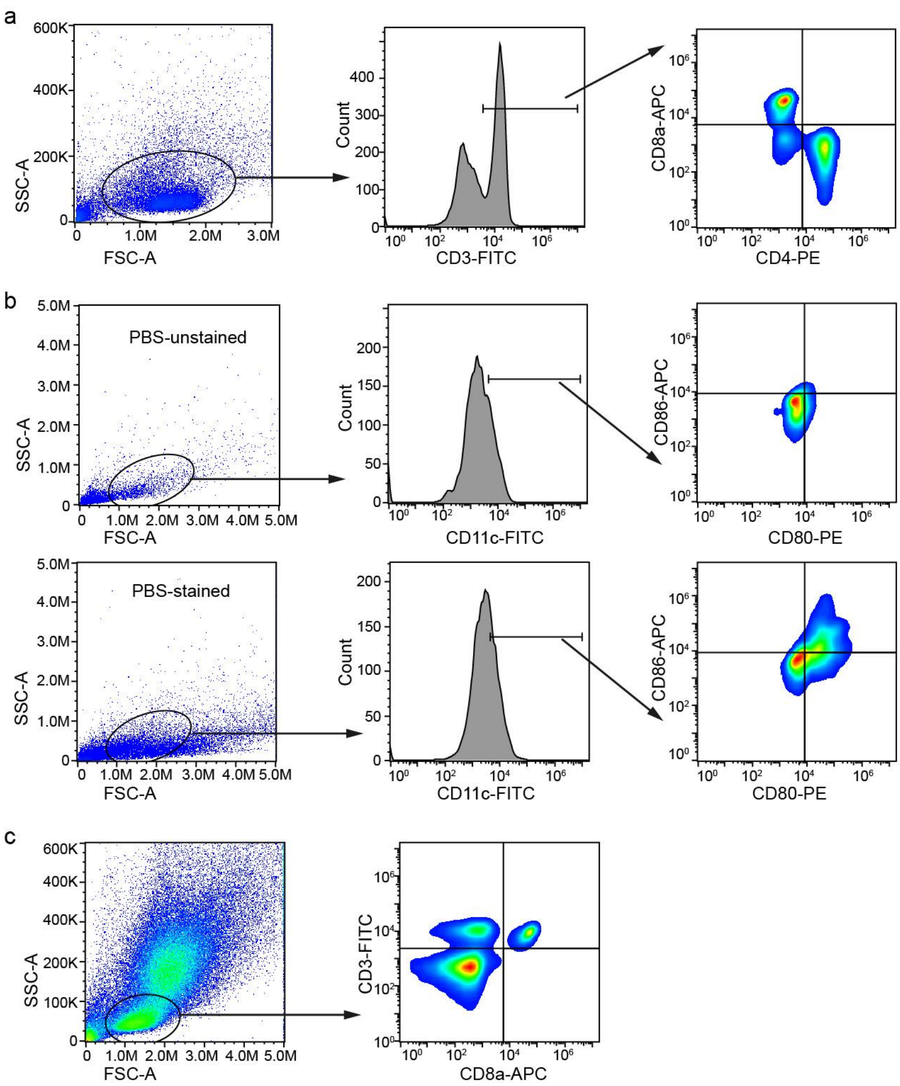

Supplementary Figure 7│Flow cytometry sequential gating strategies. a Gating strategy to determine the percentage of activated T cells presented in Fig. 3b and Fig. 3g. **b** Gating strategy to determine the percentage of activated DCs presented in Fig. 3c and Supplementary Fig. 8. **c** Gating strategy to determine the percentage of CD3+CD8+ T cells presented in Fig. 5h.

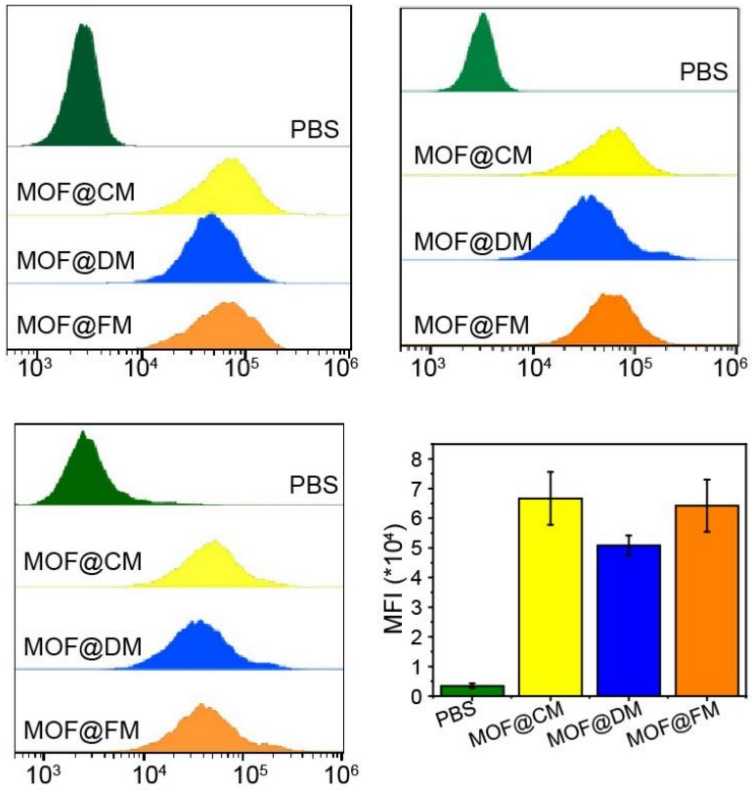

Supplementary Figure 8│Flow cytometry analysis of the uptake of BMDCs to MOF@CM, MOF@DM and MOF@FM. Flow cytometry analysis of the uptake of BMDCs to MOF@CM, MOF@DM and MOF@FM and the corresponding mean fluorescence intensity (MFI) analysis. The mean values and s.d. were presented and measurements were taken from distinct samples (n = 3). Source data are provided as a Source Data file.

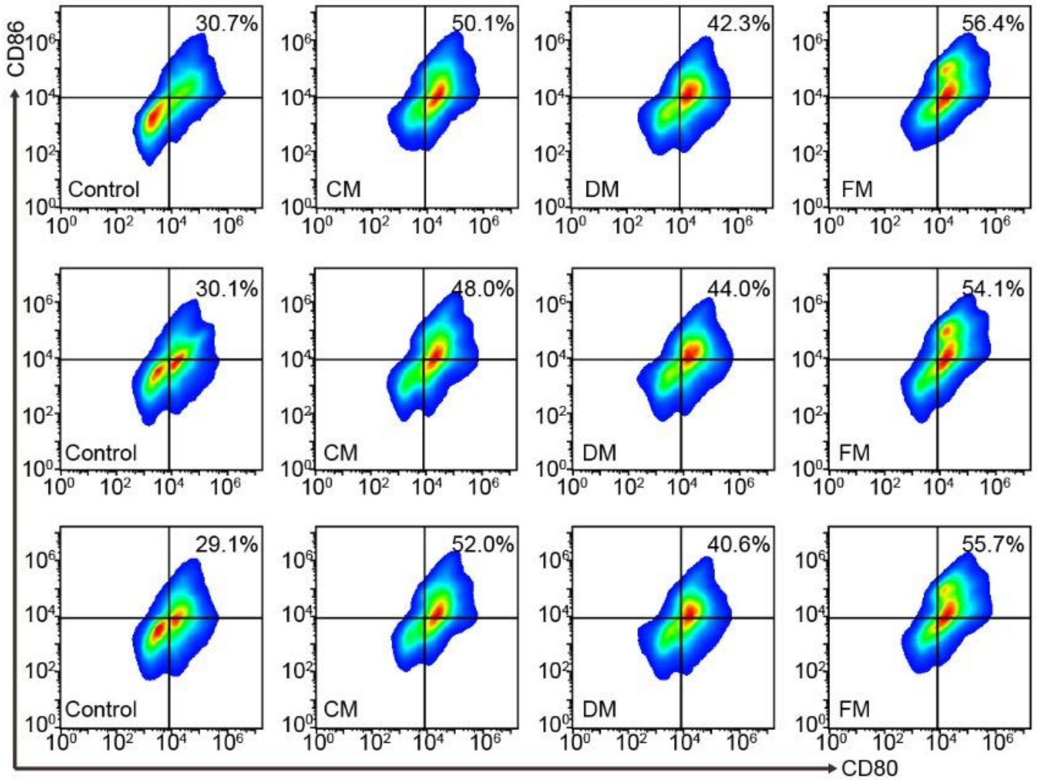

Supplementary Figure 9│DCs maturation after treatment with CM, DM and FM, respectively. Flow cytometric quantification of the expression of CD80 and CD86 (the markers for DC maturation) after in vitro incubation of DCs with CM, DM and FM for 48 h.

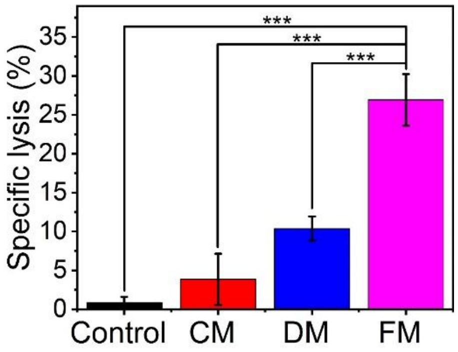

Supplementary Figure 10│In vitro cytotoxicity of the CM, DM or FM-pretreated DCs activating T lymphocytes against 4T1 cells. The mean values and s.d. were presented and measurements were taken from distinct samples. (one-way ANOVA; ** $p<0.01$, *** $p<0.001$, $n = 5$). Source data are provided as a Source Data file.

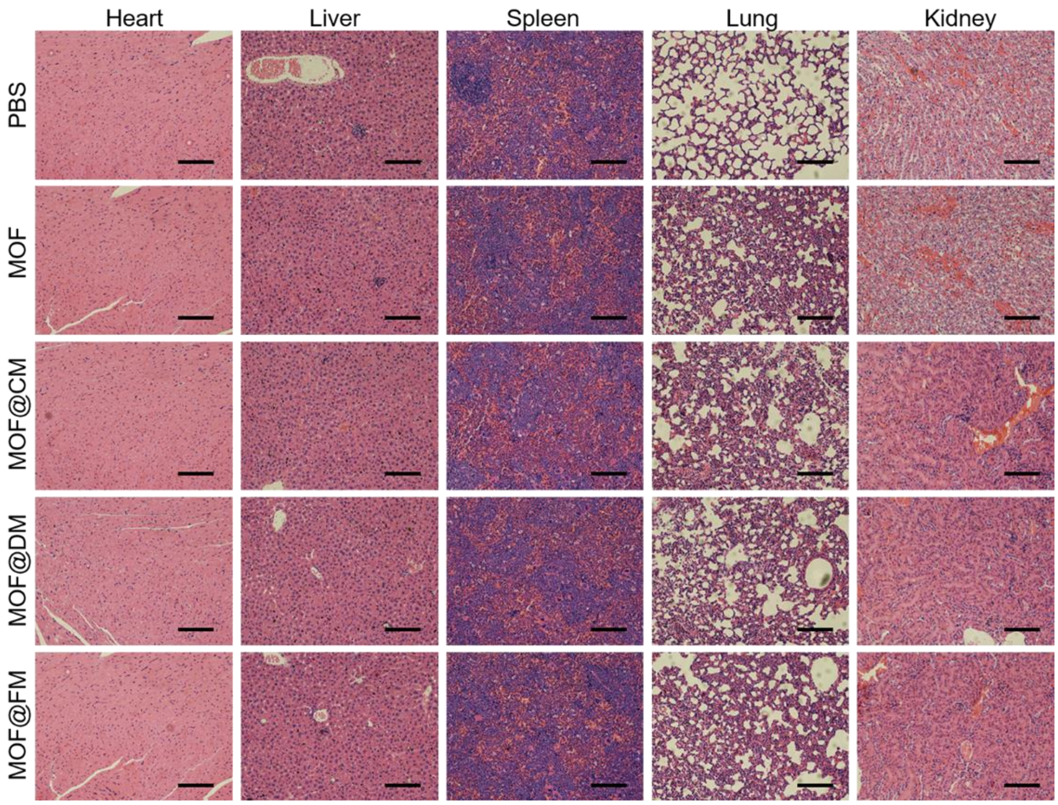

Supplementary Figure 11│H&E staining of major organs at 36 d after tumor inoculation. Scale bar = 20 μm.

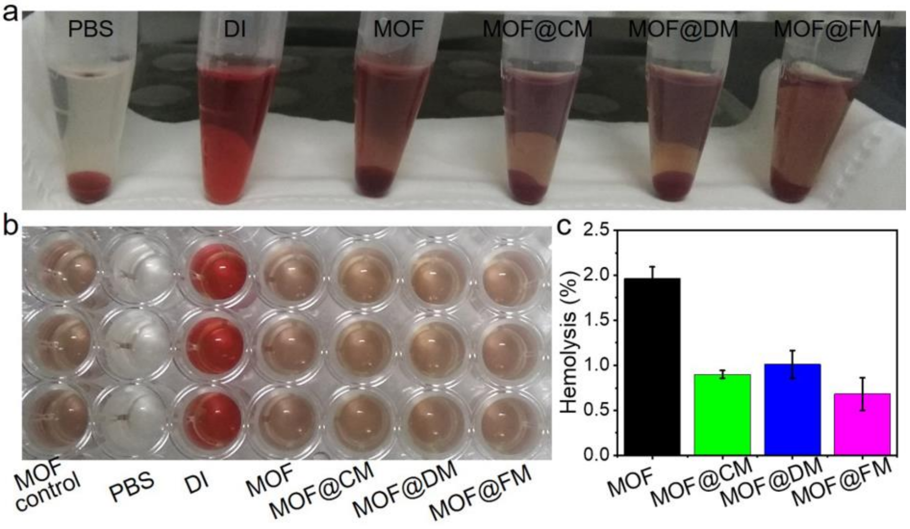

Supplementary Figure 12│Hemolytic assay of MOF, MOF@CM, MOF@DM and MOF@FM. a The hemolytic image (from left to right: PBS, deionized water (DI), MOF, MOF@CM, MOF@DM and MOF@FM) in tubes and (**b**) plates (MOF control was used to deduct the absorbance of MOF). **c** the hemolysis analysis of MOF, MOF@CM, MOF@DM and MOF@FM. The mean values and s.d. were presented and measurements were taken from distinct samples (n = 3). Source data are provided as a Source Data file.
