# Supplementary material for: Cytomembrane nanovaccines show therapeutic effects by mimicking tumor cells and antigen presenting cells
Source: Nat Commun. 2019 Jul 19;10:3199. doi: 10.1038/s41467-019-11157-1 (PMC6642123; doi:10.1038/s41467-019-11157-1)
Supplement: Supplementary file 2 — Reporting Summary [file 41467_2019_11157_MOESM2_ESM.pdf]

nature research

Corresponding author(s): Jun Feng, NCOMMS-19-02070B

Last updated by author(s): May 24, 2019

Reporting Summary

Nature Research wishes to improve the reproducibility of the work that we publish. This form provides structure for consistency and transparency in reporting. For further information on Nature Research policies, see Authors & Referees and the Editorial Policy Checklist.

Statistics

For all statistical analyses, confirm that the following items are present in the figure legend, table legend, main text, or Methods section.

n/a	Confirmed	
☐	☒	The exact sample size (n) for each experimental group/condition, given as a discrete number and unit of measurement
☐	☒	A statement on whether measurements were taken from distinct samples or whether the same sample was measured repeatedly
☐	☒	The statistical test(s) used AND whether they are one- or two-sided<br>*Only common tests should be described solely by name; describe more complex techniques in the Methods section.*
☒	☐	A description of all covariates tested
☒	☐	A description of any assumptions or corrections, such as tests of normality and adjustment for multiple comparisons
☐	☒	A full description of the statistical parameters including central tendency (e.g. means) or other basic estimates (e.g. regression coefficient) AND variation (e.g. standard deviation) or associated estimates of uncertainty (e.g. confidence intervals)
☒	☐	For null hypothesis testing, the test statistic (e.g. F, t, r) with confidence intervals, effect sizes, degrees of freedom and P value noted<br>*Give P values as exact values whenever suitable.*
☒	☐	For Bayesian analysis, information on the choice of priors and Markov chain Monte Carlo settings
☒	☐	For hierarchical and complex designs, identification of the appropriate level for tests and full reporting of outcomes
☒	☐	Estimates of effect sizes (e.g. Cohen's d, Pearson's r), indicating how they were calculated

Our web collection on statistics for biologists contains articles on many of the points above.

Software and code

Policy information about availability of computer code

Data collection	Only commercial softwares were used to collect the data in the study, such as Zetasizer software 7.11, PerkinElmer UV WinLab 6.0.4.0738, BD Accuri C6 software 1.0.264.21, and SoftMax Pro7.0.
Data analysis	Only commercial softwares (e.g. OriginPro 2018b (64-bit) b9.5.5.409, IBM SPSS Statistics 25, and FlowJo VX.07) were used to analyse the data in the study.

For manuscripts utilizing custom algorithms or software that are central to the research but not yet described in published literature, software must be made available to editors/reviewers. We strongly encourage code deposition in a community repository (e.g. GitHub). See the Nature Research guidelines for submitting code & software for further information.

Data

Policy information about availability of data

All manuscripts must include a data availability statement. This statement should provide the following information, where applicable:

- Accession codes, unique identifiers, or web links for publicly available datasets
- A list of figures that have associated raw data
- A description of any restrictions on data availability

The data that support the findings of this study are available from the corresponding authors upon reasonable request.

Field-specific reporting

Please select the one below that is the best fit for your research. If you are not sure, read the appropriate sections before making your selection.

☒ Life sciences ☐ Behavioural & social sciences ☐ Ecological, evolutionary & environmental sciences

Life sciences study design

All studies must disclose on these points even when the disclosure is negative.

Sample size	To make sure the results have significance in statistics, at least three samples were chosen. Considering the cost of experiments, no more than five samples were chosen.
Data exclusions	No data were excluded from the analyses.
Replication	We confirmed that the attempts at replication were successful.
Randomization	The used cells and animals were randomly allocated into experimental group.
Blinding	The investigators were blinded to group allocation during data collection .

Reporting for specific materials, systems and methods

We require information from authors about some types of materials, experimental systems and methods used in many studies. Here, indicate whether each material, system or method listed is relevant to your study. If you are not sure if a list item applies to your research, read the appropriate section before selecting a response.

Materials & experimental systems

n/a	Involved in the study	
☐	☒	Antibodies
☐	☒	Eukaryotic cell lines
☒	☐	Palaeontology
☐	☒	Animals and other organisms
☒	☐	Human research participants
☒	☐	Clinical data

Methods

n/a	Involved in the study	
☒	☐	ChIP-seq
☐	☒	Flow cytometry
☒	☐	MRI-based neuroimaging

Antibodies

Antibodies used	APC anti-mouse CD44, anti-MHC II-FITC, anti-CD11c-FITC, anti-CD80-PE, anti-CD86-APC, anti-CD3-FITC, anti-CD4-PE and anti-CD8-APC antibodys are used for flow cytometric analysis and are provided by BioLegend, Inc (USA). The catalog number of APC anti-mouse CD44 antibody is 103012 and its clone is IM7. The catalog number of FITC anti-mouse MHC II antibody is 114406 and its clone is 25-9-17. The catalog number of FITC anti-mouse CD11c antibody is 117306 and its clone is N418. The catalog number of PE anti-mouse CD80 antibody is 104708 and its clone is 16-10A1. The catalog number of APC anti-mouse CD86 antibody is 105012 and its clone is GL-1. The catalog number of FITC anti-mouse CD3 antibody is 100204 and its clone is 17A2. The catalog number of PE anti-mouse CD4 antibody is 100408 and its clone is GK1.5. The catalog number of APC anti-mouse CD8a antibody is 100712 and its clone is 53-6.7.
Validation	The antibodys were used for flow cytometric analysis of antibody surface-stained cells.

Eukaryotic cell lines

Policy information about cell lines

Cell line source(s)	4T1, 3T3 and CT26 cells are all from mouse.
Authentication	4T1, 3T3 and CT26 are obtained from China Center For Type Culture Collection (CCTCC).
Mycoplasma contamination	The cell lines were not tested for mycoplasma contamination.
Commonly misidentified lines (See ICLAC register)	No commonly misidentified lines were used in this study.

Animals and other organisms

Policy information about studies involving animals; ARRIVE guidelines recommended for reporting animal research

Laboratory animals	BALB/C mice were used as laboratory animals in our paper. The mice were female and about 6 weeks.

Wild animals	No wild animals were used.
Field-collected samples	Laboratory animals were fed in Department of Chemistry, Wuhan University, Wuhan 430072, P.R. China and the room number is 514. The room temperature is about 24 degree centigrade and the photoperiod is simulating the natural photoperiod.
Ethics oversight	All of the animal experiments were conducted under protocols approved by the Institutional Animal Care and Use Committee (IACUC) of the Animal Experiment Center of Wuhan University (Wuhan, China). All mouse experimental procedures were performed in accordance with the Regulations for the Administration of Affairs Concerning Experimental Animals approved by the State Council of People's Republic of China.

Note that full information on the approval of the study protocol must also be provided in the manuscript.

Flow Cytometry

Plots

Confirm that:

☒ The axis labels state the marker and fluorochrome used (e.g. CD4-FITC).

☒ The axis scales are clearly visible. Include numbers along axes only for bottom left plot of group (a 'group' is an analysis of identical markers).

☒ All plots are contour plots with outliers or pseudocolor plots.

☒ A numerical value for number of cells or percentage (with statistics) is provided.

Methodology

Sample preparation	Generation of BMDCs. BMDCs were generated from the bone mesenchymal stem cells (BMSCs) by induced differentiation65. Briefly, the BMSCs were cultured in the RPMI-1640 medium containing 20% FBS in the presence of recombinant GM-CSF (20 ng mL-1) and IL-4 (10 ng mL-1). After 6 d, BMDCs were harvested for further use.
Instrument	TEM photos were gained from JEM-2100 (JEM Ltd., Japan). Hydrodynamic diameter and zeta potential were measured by dynamic light scattering (DLS) of Malvern Zetasizer ZEN3600. UV-vis absorbance was measured by UV-vis spectrophotometry Lambda 35 (Perkin-Elmer). The fusion of DC and 4T1 cells was performed by CLSM (PerkinElmer Ultra VIEW VoX). The flow cytometric analysis was performed by flow cytometer (BD Accuri C6).
Software	BD Accuri C6 software 1.0.264.21 was used to collect flow cytometry data and FlowJo VX.07 was used to analyse flow cytometry data.
Cell population abundance	By stained with specific fluorescence-labeled antibodys, cells were separated into different parts and FlowJo can calculate the fractions.
Gating strategy	Anti-CD11c-FITC antibody was used to maker dendritic cells, and Anti-CD3-FITC antibody was used to label T cells. PBS-treated cells were stained with/without Anti-CD11c-FITC antibody or Anti-CD3-FITC antibody to determine gate.

☒ Tick this box to confirm that a figure exemplifying the gating strategy is provided in the Supplementary Information.
